# Ambulatory management of acute uncomplicated diverticulitis (AmbUDiv study): a multicentre, propensity score matching study

**DOI:** 10.1007/s00384-024-04759-9

**Published:** 2024-11-18

**Authors:** Ali Yasen Mohamedahmed, Mohammed Hamid, Mohamed Issa, Mohamed Albendary, Emiko Sultana, Shafquat Zaman, Santosh Bhandari, Diwakar Sarma, William Ball, Pradeep Thomas, Najam Husain

**Affiliations:** 1 Department of General and Colorectal Surgery, University Hospitals of Derby and Burton NHS Foundation Trust, Queen’s Hospital Burton, Burton on Trent, UK; 2 Department of General Surgery, Wye Valley NHS Trust, Hereford County Hospital, Hereford, UK; 3 Department of General and Colorectal Surgery, Sandwell and West Birmingham NHS Trust, Birmingham, UK; 4 Department of General Surgery, North West Anglia NHS Trust, Peterborough, UK; 5 Department of General and Colorectal Surgery, Shrewsbury and Telford Hospital NHS Trust, Shrewsbury, UK; 6 College of Medical and Dental Science, School of Medicine, University of Birmingham, Edgbaston, Birmingham, UK

**Keywords:** Uncomplicated diverticulitis, Hinchey 1a, Ambulatory management

## Abstract

**Introduction:**

Recent studies have suggested that ambulatory management is feasible for acute uncomplicated diverticulitis (AUD); however, there is still no consensus regarding the most appropriate management settings. This study presents a multi-centre experience of managing patients presenting with AUD, specifically focusing on clinical outcomes and comparing ambulatory treatment with in-patient management.

**Methods:**

A retrospective multi-centre study was conducted across four hospitals in the UK and included all adult patients with computed tomography (CT) confirmed (Hinchey grade 1a) acute diverticulitis over a 12-month period (January – December 2022). Patient medical records were followed up for 1-year post-index episode, and outcomes were compared between those treated through the ambulatory pathway versus inpatient treatment using 1:1 propensity score matching (PSM). All statistical analysis was performed using the R Foundation for Statistical Computing, version 4.4.

**Results:**

A total of 348 patients with Hinchey 1a acute diverticulitis were included (260 in-patients; 88 ambulatory pathway), of which nearly a third (31.3%) had a recurrent disease. Inpatient management was dominant (74.7%), with a median of 3 days of hospital stay. PSM resulted in 172 patients equally divided between the two care settings. Ambulatory management was associated with a lower readmission rate (*P* = 0.02 before PSM, *P* = 0.08 after PSM), comparable surgical (*P* = 0.57 before PSM, 0% in both groups after PSM) and radiological interventions (*P* = 0.99 before and after PSM) within one year. In both matched and non-matched groups, a strong association between readmissions and inpatient management was noted in univariate analysis (*P* = 0.03 before PSM, *P* = 0.04 after PSM) and multivariate analysis (*P* = 0.02 before PSM, *P* = 0.03 after PSM).

**Conclusion:**

Our study supports the safety and efficacy of managing patients with AUD through a well-designed ambulatory care pathway. In particular, hospital re-admission rates are lower and other outcomes are non-inferior to in-patient treatment. This has implications for substantial cost-savings and better utilisation of limited healthcare resources.

**Supplementary information:**

The online version contains supplementary material available at 10.1007/s00384-024-04759-9.

## Introduction

Diverticular disease is common, with an estimated prevalence of up to 85% in patients aged 50 years and above and a 4% lifetime risk of developing acute diverticulitis [1, 2]. The increasing occurrence within Western populations incurs a significant burden on patient quality of life and the economy worldwide. In the United States, the disease is thought to adversely impact the economy to an estimated two billion dollars per annum in direct and indirect costs [3]. In the United Kingdom, studies have reported up to 5% consumption of annual general surgical budgets being utilised for investigations and management of this condition [4]. 

A significant contribution to cost and quality of life is the in-patient management of diverticular disease. This remains a popular choice amongst clinicians despite 80% of patients presenting with uncomplicated diverticulitis [5]. Traditionally, hospitalisation offers bed rest, analgesia, replacement of electrolytes, and treatment with intravenous fluid and antibacterials.

Recent prospective trials have suggested that ambulatory management is non-inferior to in-patient admission and treatment of uncomplicated diverticulitis with regard to re-admission and recurrence rates, with substantial cost savings for healthcare providers (approximately three times lower) [6]. In such cases (Hinchey grade 1a), the adoption of a no-antibiotic strategy provides a shorter duration of treatment time and a lower disease recurrence rate. Moreover, no difference was observed in short-term morbidity and mortality and the need for elective or emergency resections between the in-patient and ambulatory cohorts [5, 7]. Additionally, the rate of treatment failure in the outpatient setting appears not to be influenced by previous episodes of acute diverticulitis, the type of antibiotic treatment prescribed or the presence of co-morbidities [8]. 

The present literature and reviews have some methodological limitations, with low-quality studies and few prospective, randomised trials included. There remains no consensus on the in-patient versus ambulatory management of acute uncomplicated diverticulitis (AUD) [9]. 

This study aims to report primary and secondary outcomes of four hospitals’ experiences in managing AUD in an ambulatory setting compared with in-patient treatment. To our knowledge, this is the first study to explore ambulatory treatment for AUD at a multicentre level in the UK.

## Methods

### Study design and setting

This was a retrospective multicentre study conducted across four hospitals in the United Kingdom (Queen’s Hospital Burton, Sandwell General Hospital, Peterborough City Hospital, and Royal Shrewsbury Hospital). The study was approved by the local audit department of each participating hospital and was conducted and reported in accordance with the Declaration of Helsinki and STROBE recommendations, respectively (Appendix 1). All patients presenting with computed tomography (CT) confirmed acute diverticulitis from 01/01/2022 till 31/12/2022 were considered against the study inclusion and exclusion criteria. All included patients had a follow-up period of at least 12 months.

### Patient selection criteria

Eligible participants were identified through each hospital’s electronic medical records. A modified Hinchey classification system was used to grade the severity of diverticulitis [10]. Our inclusion criteria were adult patients (aged ≥ 18 years) presenting with abdominal pain and a CT scan showing colonic Hinchey Ia acute diverticulitis. Hinchey Ia was defined as confined/localised pericolic inflammation (phlegmon).

Patients with diverticulitis in the gastrointestinal tract other than the colon, cases of complicated diverticulitis (Hinchey grade Ib: confined pericolic abscess, grade II: pelvic, intraabdominal, or retroperitoneal abscess; grade III: generalised purulent peritonitis; and grade IV: faecal peritonitis) and patients diagnosed clinically in the absence of radiological confirmation (no CT scan) were excluded. Moreover, patients with incomplete datasets or equivocal CT findings were also excluded.

### Data collection

Data were extracted from all eligible patients’ electronic and (where necessary) archived medical case notes and stored on an encrypted, password-protected computer. Data included demographics (age, gender, comorbidities (including previous episodes of diverticulitis), and steroid use), vital signs scores (temperature (°C)), respiratory rate, blood pressure (mmHg), and levels of consciousness assessed using the Glasgow Coma Scale (GCS), inflammatory markers on presentation (White Blood Cell Count (WCC, x 10*9/L) and C-Reactive Protein (CRP, mg/L)), antibiotic prescription, treatment setting (ambulatory vs. inpatient), length of hospital stay (LOS) and follow-up colonic investigation(s).

Additionally, all patients were followed up for 12 months after the index AUD presentation, and any surgical or radiological intervention(s) were recorded. In this study, ambulatory care was defined as < 24-hour hospital stay. Patients staying in the hospital for ≥ 24 h were categorised as in-patients.

### Outcomes

Our primary outcome of interest was hospital admission with acute diverticulitis within 12 months of the index presentation. This was defined as patients presenting with symptoms typical of acute diverticulitis necessitating admission to an in-patient bed for further observation and treatment. These included either patients presenting with new symptoms (recurrent disease/flare-up) or treatment failure of the initial episode.

Our analysed secondary outcomes were as follows: any surgical or radiological intervention for diverticulitis during the follow-up period, LOS (in days), post-recovery colonic investigation(s) (colonoscopy, flexible sigmoidoscopy, CT colonography), and a diagnosis of colorectal cancer (CRC) within the first year.

### Statistical analysis

The overall unmatched and matched data were analysed. Two propensity score-matched (PSM) groups of near-equal size for ambulatory versus inpatient management of Hinchey 1a diverticulitis were formed. PSM was performed using one-on-one near-neighbour matching with a calliper of 0.1. The variables used for matching were age, gender, comorbidities (previous diverticulitis diagnosis, diabetes mellitus, and steroid usage), inflammatory markers on presentation (WCC and CRP), and antibiotic treatment. A standardised mean difference (SMD) of less than 0.1 was deemed negligible, indicating appropriate matching (Appendix 2).

Data are summarised using median and interquartile range (IQR) for continuous variables and number and percentage for categorical data. Variables are compared between ambulatory-care and inpatient-care groups using the Mann-Whitney U tests for continuous variables and Fisher’s exact analysis for categorical variables. Odds ratio (OR) and 95% confidence intervals (95% CI) were calculated for patient factors and outcomes associated with the ambulatory care of Hinchey 1a diverticulitis, using both univariate and multivariate binomial logistic regression (adjusting for age, gender and comorbidities). P-values of < 0.05 were considered statistically significant. Statistical analysis was performed using R version 4.4 (R Foundation for Statistical Computing, Vienna, Austria) utilising the matchit, haven and cobalt packages.

## Results

### Patient characteristics

**Fig. 1:**
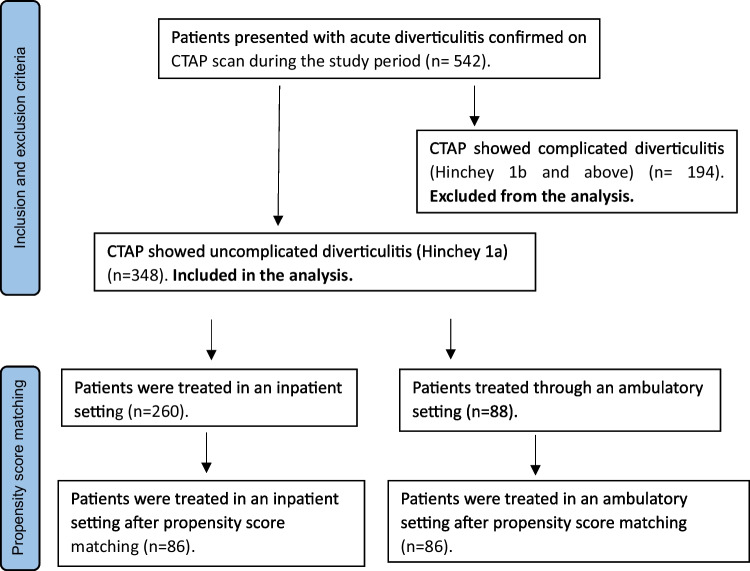
Study flow diagram. CTAP: computed tomography of abdomen and pelvis, n: total number of patients

A total of 348 patients with Hinchey 1a acute diverticulitis presenting during the study period were included. The majority of these were treated as in-patients [*n* = 260/348, (74.7%)], and the remainder [*n* = 88/348, (25.3%)] in an ambulatory setting. Figure 1 demonstrates the study flow diagram, and patient characteristics are summarised in Table 1.

**Table 1:** Comparison of patient’s characteristics and treatment outcomes between ambulatory and inpatient groups before and after propensity score matching (PSM)

Characteristic	Before PSM matching				After PSM matching			
	All (*N* = 348)	Ambulatory (*n* = 88)	Inpatient (*n* = 260)	*P*-value	All (*N* = 172)	Ambulatory (*n* = 86)	Inpatient (*n* = 86)	*P*-value
Age, median (IQR) years	62 (51–74)	59 (50–72)	62 (51–75)	0.153	62 (52–72)	59 (51–72)	63 (53–73)	0.307
Female gender, n (%)	228 (65.5)	58 (65.9)	170 (65.4)	> 0.999	113 (65.7)	57 (66.3)	56 (65.1)	> 0.999
Diabetic, n (%)	40 (11.5)	6 (6.8)	34 (13.1)	0.125	14 (8.1)	6 (7.0)	8 (9.3)	0.782
Use of steroids, n (%)	14 (4.0)	1 (1.1)	13 (5.0)	0.205	2 (1.2)	1 (1.2)	1 (1.2)	> 0.999
Previous diverticulitis, n (%)	109 (31.3)	23 (26.1)	86 (33.1)	0.235	45 (26.2)	23 (26.7)	22 (25.0)	> 0.999
Complicated	23 (6.6)	4 (4.6)	19 (7.3)	0.463	5 (2.9)	4 (4.7)	1 (1.2)	0.368
Vital signs, n (%)								
Temperature ≥ 38 °C	18 (5.2)	1 (1.1)	17 (6.5)	0.052	5 (2.9)	1 (1.2)	4 (4.7)	0.368
Elevated RR ≥ 20	14 (4.0)	1 (1.1)	13 (5.0)	0.205	5 (2.9)	1 (1.2)	4 (4.7)	0.368
Low SBP ≤ 90 mmHg	19 (5.5)	2 (2.3)	17 (6.5)	0.176	9 (5.2)	2 (2.3)	7 (8.1)	0.168
Altered GCS < 15	1 (0.3)	0 (0.0)	1 (0.4)	> 0.999	1 (0.6)	0 (0.0)	1 (1.2)	> 0.999
Inflammatory markers, n (%)								
WCC (x 10*9/L)								
<10	119 (34.2)	32 (36.4)	87 (33.5)	0.697	65 (37.8)	31 (36.1)	34 (39.5)	0.753
10–15	160 (46.0)	50 (56.8)	110 (42.3)	0.019*	88 (51.2)	49 (57.0)	39 (45.4)	0.170
15–20	57 (16.4)	6 (6.8)	51 (19.6)	0.004*	18 (10.5)	6 (7.0)	12 (14.0)	0.212
> 20	12 (3.4)	0 (0.0)	12 (4.6)	0.042*	1 (0.6)	0 (0.0)	1 (1.2)	> 0.999
CRP (mg/L)								
< 50	155 (44.5)	49 (55.7)	106 (40.8)	0.018*	95 (55.2)	47 (54.7)	48 (55.8)	> 0.999
50–100	72 (20.7)	14 (15.9)	58 (22.3)	0.226	37 (21.5)	14 (16.3)	23 (26.7)	0.137
100–200	101 (29.0)	24 (27.3)	77 (29.6)	0.786	37 (21.5)	24 (27.9)	13 (15.1)	0.063
200–300	14 (4.0)	0 (0.0)	14 (5.4)	0.025*	2 (1.2)	0 (0.0)	2 (2.3)	0.497
>300	6 (1.7)	1 (1.1)	5 (1.9)	> 0.999	1 (0.6)	1 (1.2)	0 (0.0)	> 0.999
Antibiotics prescription n (%)	337 (96.8)	83 (94.3)	254 (97.7)	0.154	164 (95.3)	83 (96.5)	81 (94.2)	0.720
Length of stay, median (IQR) days	2 (0–3)	0 (0–0)	3 (2–4)	< 0.001†	0.5 (0–2)	0 (0–0)	2 (1–4)	< 0.001†
Re-admission within 1-year, n (%)	63 (18.1)	9 (10.2)	54 (20.8)	0.026*	25 (14.5)	8 (9.3)	17 (19.8)	0.082
IR within 1-year, n (%)	1 (0.3)	0 (0.0)	1 (0.4)	> 0.999	1 (0.6)	0 (0.0)	1 (1.2)	> 0.999
Surgery within 1-year, n (%)	3 (0.9)	0 (0.0)	3 (1.2)	0.575	0 (0.0)	0 (0.0)	0 (0.0)	-
CRC within 1-year, n (%)	5 (1.4)	2 (2.3)	3 (1.2)	0.604	2 (1.2)	2 (2.3)	0 (0.0)	0.497
1-year follow up, n (%)	188 (54.0)	51 (58.0)	137 (52.7)	0.458	97 (56.4)	50 (58.1)	47 (54.7)	0.759
Endoscopy, n (%)	176 (50.6)	46 (52.3)	130 (50.0)	0.805	89 (51.7)	45 (52.3)	44 (51.2)	> 0.999
Weeks post-review, median (IQR)	7 (6–11)	6 (5.8–8)	8 (6–12)	0.055	7 (6–11)	6 (6–8)	8 (6–12)	0.089
Flexible sigmoidoscopy, n (%)	86 (24.7)	23 (26.1)	63 (24.2)	0.775	41 (23.8)	23 (26.7)	18 (20.9)	0.474
Colonoscopy, n (%)	90 (25.9)	23 (26.1)	67 (25.8)	> 0.999	48 (27.9)	22 (25.6)	26 (30.2)	0.610
CTC, n (%)	15 (4.3)	7 (8.0)	8 (3.1)	0.067	10 (5.8)	7 (8.1)	3 (3.5)	0.329
Weeks before CTC	9.5 (6–27.3)	8.5 (5.5–24.8)	9.5 (6–46)	0.423	2 (1–2)	2 (1–2)	2 (1–4)	0.617

*Indicates statistically significant using Fisher’s exact analysis

†Indicates statistically significant using Mann-Whitney U-test

*IQR* interquartile range, *RR* Respiratory rate, *SBP *Systolic blood pressure,* GCS* Glasgow coma scale, *WCC* White cell count, *CRP* C-reactive protein, *IR* Interventional radiology, *CRC* Colorectal cancer, *CTC* Computed tomography colonography

The median age of our cohort was 62 years (51–74), and 228 (65.5%) were female. Approximately one-third (*n* = 109, (31.3%)) had a previous diagnosis of diverticulitis, and of these patients, 23 (6.6%) were graded as having complicated disease. Forty patients (11.5%) were co-morbid with diabetes mellitus, and 14 (4.0%) were using long-term steroids.

On presentation, 18 (5.2%) patients had a fever (temperature ≥ 38 °C), 14 (4.0%) were tachypnoeic (RR ≥ 20), 19 (5.5%) were hypotensive (systolic blood pressure ≤ 90 mmHg), and one (0.3%) had reduced level of consciousness (GCS < 15).

One-to-one PSM yielded a cohort of 172 patients, of which half (*n* = 86) were treated in the ambulatory setting and the other half (*n* = 86) as in-patients. The median age of this group was 62 years (52–72), and 113 (65.7%) were female.

Of the total cohort, 45 (26.2%) patients had a previous diagnosis of diverticulitis, and 5 (2.9%) of these were classified as complicated disease. On presentation, 5 (2.9%) patients had a fever (temperature ≥ 38 °C), 5 (2.9%) were tachypnoeic (RR ≥ 20), 9 (5.2%) were hypotensive (systolic blood pressure ≤ 90 mmHg), and one (0.6%) had an altered level of consciousness (GCS < 15).

Patients presenting with a raised temperature (≥ 38 °C) were significantly less likely to be managed in the ambulatory setting in adjusted models for the unmatched data [OR 0.16 (0.02, 1.25), *P* = 0.023], becoming less significant on matched data [OR 0.22 (0.02, 1.98), *P* = 0.128] (Table 2).

**Table 2:** Patient factors associated with ambulatory care using univariate and multivariate binomial logistic regression modelling for the unmatched data

Characteristic	Univariate OR (95% CI)	*p*-value	Multivariate OR (95% CI)	*p*-value
Age	1.00 (0.97, 1.03)	0.871	1.00 (0.96, 1.04)	0.871
Female gender	0.72 (0.27, 1.92)	0.548	0.73 (0.26, 2.04)	0.551
Diabetic	0.76 (0.15, 3.75)	0.751	0.77 (0.15, 3.84)	0.745
Steroid use	0.98 (0.10, 9.20)	0.951	1.07 (0.11, 10.83)	0.952
Previous diverticulitis	0.72 (0.42, 1.23)	0.312	0.75 (0.44, 1.30)	0.307
Complicated	0.78 (0.24, 2.59)	0.665	0.76 (0.22, 2.60)	0.660
Temperature > 38 °C	0.16 (0.02, 1.25)	0.081	0.16 (0.02, 1.25)	0.023*
Elevated RR	0.22 (0.03, 1.69)	0.161	0.23 (0.03, 1.80)	0.088
Low SBP	0.33 (0.08, 1.47)	0.223	0.39 (0.09, 1.77)	0.176
Altered GCS	0.00 (0.00, INF)	0.988	0.00 (0.00, INF)	0.419
WCC 10–15 (x 10*9/L)	1.79 (1.1, 2.92)	0.032*	1.71 (1.05, 2.80)	0.031*
WCC > 15 (x 10*9/L)	0.23 (0.10, 0.55)	0.003*	0.27 (0.11, 0.64)	< 0.001*
CRP < 50 (mg/L)	1.83 (1.12, 2.97)	0.043*	1.67 (1.02, 2.73)	0.042*
CRP > 200 (mg/L)	0.15 (0.02, 1.11)	0.105	0.18 (0.02, 1.42)	0.040*

*Indicates statistically significant

95% *CI* 95% confidence interval, *RR* Respiratory rate, *SBP *Systolic blood pressure, *GCS* Glasgow coma scale, *WCC* White cell count, *CRP *C-reactive protein

### Inflammatory markers and management decision

In unmatched data, patients were more likely to be treated in the ambulatory care setting if their WCC was in the range of 10–15 × 10*9/L and a CRP of less than 50 mg/L. Patients were more likely to be admitted to a hospital bed if their WCC was more than 15 × 10*9/L and CRP was greater than 200 mg/L (Table 1).

These findings were also valid in the multivariate adjusted models associated with ambulatory care for the unmatched data [WCC 10–15 × 10*9/L: OR 1.71 (1.05, 2.80), P *=* 0.031], [CRP < 50 mg/L, OR 1.67 (1.02, 2.73), *P* = 0.042], [WCC > 15 × 10*9/L: OR 0.27 (0.11, 0.64), P *<* 0.001], [CRP > 200 mg/L: OR 0.18 (0.02, 1.42), *P* = 0.040] (Table 2). The statistical significance of these parameters was lost in the matched data (Table 1).

However, in the multivariate adjusted models associated with ambulatory care for the matched data, CRP of < 200 mg/L remained significantly associated with treatment in the ambulatory setting [CRP < 200 mg/L, OR2.72 (1.21, 6.12), *P* = 0.012] (Table 3).

**Table 3:** Patient factors associated with ambulatory care using univariate and multivariate binomial logistic regression modelling for the matched data

Characteristic	Univariate OR (95% CI)	*p*-value	Multivariate OR (95% CI)	*p*-value
Age	0.99 (0.97, 1.01)	0.349	0.99 (0.97,1.01)	0.348
Female gender	1.05 (0.56, 1.98)	0.856	1.06 (0.56, 2.01)	0.856
Diabetic	0.73 (0.24, 2.20)	0.714	0.81 (0.26, 2.52)	0.713
Steroid use	1.00 (0.06, 16.25)	0.969	0.95 (0.06, 15.51)	0.969
Previous diverticulitis	1.06 (0.54, 2.10)	0.702	1.15 (0.57, 2.32)	0.702
Complicated	4.67 (0.48, 45.62)	0.200	4.67 (0.44, 49.21)	0.161
Temperature > 38 °C	0.24 (0.03, 2.20)	0.175	0.22 (0.02,1.98)	0.128
Elevated RR	0.24 (0.03, 2.20)	0.238	0.26 (0.03,2.44)	0.194
Low SBP	0.27 (0.05, 1.33)	0.116	0.27 (0.05, 1.38)	0.088
Altered GCS	0.00 (0.00, INF)	0.987	0.00 (0.00, INF)	0.216
WCC 10–15 (x 10*9/L)	1.6 (0.87, 2.91)	0.139	1.58 (0.86, 2.89)	0.138
WCC > 15 (x 10*9/L)	0.42 (0.15, 1.17)	0.148	0.47 (0.17, 1.31)	0.138
CRP < 200 (mg/L)	2.17 (1.02, 4.63)	0.016*	2.72 (1.21, 6.12)	0.012*

*Indicates statistically significant

95% *CI*: 95% confidence interval, *RR* Respiratory rate, *SBP* Systolic blood pressure, *GCS *Glasgow coma scale, *WCC* White cell count, *CRP* C-reactive protein

### Antibiotic usage

Antibiotics were used in the treatment of 337/348 (96.8%) patients (Table 1). Antibiotics were unlikely to be prescribed for patients with a WCC < 10 × 10*9/L and CRP < 50 mg/L in multivariate adjusted models for both the unmatched and matched data [OR 0.15 (0.04, 0.53), *P* = 0.003], [OR 0.10 (0.02, 0.54), *P* = 0.003], respectively. The likelihood of antibiotics not being prescribed dissipates with increasing WCC or CRP from the above levels.

### Length of hospital stay

Patients admitted into the hospital stayed a median of 3 (2–4) days in the unmatched cohort and 2 (1–2) days in the matched cohort, in comparison to zero days (*P* < 0.001) for corresponding patients treated via ambulatory care (Table 1). This significant difference remained in multivariate-adjusted models (Table 4 and 5).

**Table 4:** Outcomes associated with ambulatory care using univariate and multivariate binomial logistic regression models for the unmatched data

Characteristic	Univariate OR (95% CI)	*p*-value	Multivariate OR (95% CI)	*p*-value
Length of stay	0.00 (0.00, INF)	0.987	0.00 (0.00, INF)	< 0.001*
IR drainage	0.00 (0.00, INF)	0.988	0.00 (0.00, INF)	0.390
Surgery	0.00 (0.00, INF)	0.986	0.00 (0.00, INF)	0.144
Readmission	0.43 (0.20, 0.92)	0.039*	0.45 (0.21, 0.96)	0.028*
Follow up	1.21 (0.74, 1.98)	0.589	1.15 (0.70, 1.88)	0.589
Endoscopy (≤ 8 weeks)	2.40 (1.10, 5.25)	0.040*	2.31 (1.04, 5.12)	0.032*
Flexible sigmoidoscopy	1.06 (0.54, 2.08)	0.931	1.03 (0.51, 2.09)	0.931
Colonoscopy	0.94 (0.48, 1.84)	0.931	0.97 (0.48, 1.97)	0.931
CTC (≤ 8 weeks)	1.00 (0.12, 8.31)	0.864	1.22 (0.13, 11.34)	0.864
Cancer with one year	1.99 (0.33, 12.12)	0.376	2.35 (0.35, 15.62)	0.390

*Indicates statistically significant

95% *CI*: 95% confidence interval, *IR *Interventional radiology, *CTC *Computed tomography colonography

**Table 5:** Outcomes associated with ambulatory care using univariate and multivariate binomial logistic regression models for the matched data

Characteristic	Univariate OR (95% CI)	*p*-value	Multivariate OR (95% CI)	*p*-value
Length of stay	0.00 (0.00, INF)	0.999	0.00 (0.00, INF)	< 0.001*
IR drainage	0.00 (0.00, INF)	0.987	0.00 (0.00, INF)	0.232
Surgery	0.00 (0.00, INF)	0.986	0.00 (0.00, INF)	0.239
Readmission	0.42 (0.17, 1.02)	0.044*	0.39 (0.16, 0.97)	0.037*
Follow up	1.15 (0.63, 2.11)	0.655	1.15 (0.62, 2.12)	0.655
Endoscopy (≤ 8 weeks)	2.42 (0.96, 6.11)	0.051	2.52 (1.00, 6.37)	0.047*
Flexible sigmoidoscopy	1.51 (0.65, 3.49)	0.419	1.42 (0.60, 3.35)	0.418
Colonoscopy	0.66 (0.29, 1.53)	0.469	0.72 (0.30, 1.75)	0.469
CTC (≤ 8 weeks)	0.50 (0.03, 8.95)	0.621	0.46 (0.02, 9.91)	0.614
Cancer with one year	> 100 (0.00, INF)	0.988	> 100 (0.00, INF)	0.108

*Indicates statistically significant

95% *CI*: 95% confidence interval, *IR* Interventional radiology, *CTC *Computed tomography colonography

#### Re-admission with acute diverticulitis during the follow-up period (12 months)

In the unmatched cohort, patients requiring in-patient treatment were more likely to be re-admitted with diverticulitis within the first year from the index presentation (20.8% vs. 10.2%, *P* = 0.026) (Table 1). The level of significance was not equivalent in the matched cohort (19.8% vs. 9.3%, *P* = 0.082) (Table 1); however, the association of reduced readmission and prior ambulatory treatment was maintained in both unmatched and matched cohorts in adjusted models [OR 0.45 (0.21, 0.96, *p* = 0.028] (Table 4), [OR 0.39 (0.16, 0.97), *P* = 0.037] (Table 5), respectively.

#### Surgical and radiological intervention during the follow-up period (12 months)

There was a comparable frequency in the surgical and radiological interventions required by patients in both the ambulatory and in-patient groups within the first year. This was observed for both the unmatched and matched cohorts (Tables 1 and 4, 5).

### Follow-up investigations during the follow-up period (12 months)

No significant difference was detected in the number of follow-up investigations (flexible sigmoidoscopy, colonoscopy and CT colonography) performed for patients in both the ambulatory and in-patient groups within the first year. This was true for both the unmatched and matched cohorts (Tables 1 and 4, 5). However, endoscopic investigations were more likely to be performed earlier (≤ 8 weeks) for patients treated via the ambulatory care pathway, and this was seen in both unmatched and matched cohorts [OR 2.31 (1.04, 5.12), *P* = 0.032] (Table 4), [OR 2.52 (1.00, 6.37), *P* = 0.047], respectively (Table 5).

#### Colorectal cancer diagnosis during the follow-up period (12 months)

There was no significant difference in the incidence of CRC diagnosis in patients in either treatment group within the first year; before PSM (ambulatory 2.3% vs. in-patient 1.2%, P *=* 0.60) and after PSM (ambulatory 2.3% vs. 0.0% in-patient, P *=* 0.49) (Table 1). This was also true in the adjusted models for unmatched and matched cohorts (Table 4 and 5).

## Discussion

Literature and medical societies’ guidelines suggest that ambulatory treatment of AUD is safe and efficacious in more than 90% of patients presenting to hospital emergency departments [1, 6, 7, 11]. Despite these findings, in-patient management remains popular in treating this condition.

In the present study, 75% of patients with AUD were admitted to the hospital, in keeping with previous reports [6, 12]. Multivariate analysis demonstrated that abnormal vital signs and raised inflammatory markers were the main factors influencing this decision. This high inpatient management rate could be due to a lack of established hospital protocols and pathways advocating ambulatory management.

The antibiotic protocol used was similar across the four centres. First line for severe cases requiring IV antibiotics were co-amoxiclav, metronidazole, ± gentamycin. Oral co-amoxiclav was used when stepping down antibacterial therapy and in the ambulatory setting.

The overall re-admission rate for diverticulitis during the 12-month follow-up period was 18.1%. This was similar to the recurrence rate reported by the DIABOLO trial [13] and Strate et al. [14], 16.4% and 20%, respectively. A significantly higher recurrence rate was reported in the DIRECT trial (30%) [15] and the LASER trial (61%) [16]. These variations reported in the literature could be due to the length of follow-up (being five years in both the DIRECT and LASER trials) and the inclusion criteria adopted by these studies.

In this study, the ambulatory group was associated with a lower risk of re-admission with diverticulitis compared with the in-patient group. Several factors may explain this finding, including the nature of hospital treatment, often involving a period of intravenous antibiotics that may exacerbate gastrointestinal symptoms (abdominal cramps, nausea, diarrhoea), as well as the psychological trauma and emotional impact of hospitalisation influencing subsequent patient behaviour and choices. Different antibiotic regimens have been used for the included population, depending on local hospital protocols.

Since inception, the Hinchey classification and its various modifications and alterations have been used for treating acute diverticulitis based on CTAP findings [10, 17]. It grades the degree of inflammation and associated abscess formation and colonic perforation into four types to guide surgical management.

More recently the original classification system has been questioned for its application in modern day-to-day practice, especially with the introduction and availability of advanced CT imaging. Consequently, Sartelli et al. have suggested a simplified classification system grading acute diverticulitis into complicated and uncomplicated based on radiological (CT) findings [18]. 

The uncomplicated group includes localised colonic inflammation only (not extending to the peritoneum). The complicated diverticulitis group is subdivided into pericolic air bubbles or fluid, diverticular abscess formation, and free peritoneal fluid and widespread pneumoperitoneum. In addition to the simplified grading of disease, the Sartelli et al. classification also provides recommendations on management.

The evidence in the literature concerning patients’ role in treatment and management remains limited [19, 20]. Rate of hospital re-admission/re-attendance may be influenced by an individual’s past experience [21]. Patients with previous episodes of in-patient care may re-present to acute units whenever they experience symptom flare-up as opposed to those managed successfully in an ambulatory setting seeking medical help only when absolutely necessary. The PSM analysis used in our study aimed to neutralise biases of patient-related factors such as co-morbidities, age and gender. However, other factors, such as BMI and physical activity levels, were not considered.

By definition, there was a difference in LOS between our two groups. Patients managed as in-patients had a median LOS of 3 (unmatched cohort) and 2 (matched cohort) days, respectively. This is similar to previous studies [13, 22]; however, a longer LOS of six days was reported in two studies that reported data before 2013 [23, 24]. This reflects the recent paradigm shift toward earlier patient discharge.

The American Society of Colon and Rectal Surgeons (ASCRS) suggests that patients presenting with signs of peritonitis, are immunocompromised, of advanced age, or are unable to tolerate oral intake should be admitted for inpatient treatment [25]. Otherwise, acute diverticulitis can be managed on an outpatient basis, provided appropriate follow-up has been arranged. We would advocate for the same approach, especially in patients with AUD. Avoiding unnecessary hospitalisation allows for increased capacity and better utilisation of finite resources. Moreover, this approach is supported by our finding that no significant difference in surgical and/or radiological interventions is required within the first 12 months of index presentation between the two groups.

Following recovery from an episode of acute diverticulitis, patients must be followed up to exclude colonic malignancy [26, 27]. Several options with varying risks and benefits are available, including radiological (CT colonography, barium enema) and endoscopic (flexible sigmoidoscopy, colonoscopy) investigations. In this study, no significant difference was detected in the number of follow-up investigations performed between the two groups over the 12-month follow-up period. Moreover, luminal investigations were more likely to be performed earlier in patients managed through the ambulatory setting, with no significant differences in colorectal cancer detection rates.

Mortality rates in AUD are negligible with appropriate management [14]. Based on our study findings, and with the correct dietary advice, patient education, analgesia, and follow-up, AUD can be managed safely in an outpatient environment. The advantages of freeing up limited resources associated with this are clear and numerous. Although the present study does not address cost-savings directly, we would suggest the potential for significant economic benefits when adopting an ambulatory approach. An estimated 60–80% cost-saving per patient per episode has been reported in the literature [28, 29], - further emphasising the adoption of an ambulatory care strategy in cases of AUD. The DIVER trial, an RCT conducted in five Spanish tertiary care facilities, reported cost savings of €1,124 per patient [6]. 

The shift towards an ambulatory care model, not only for AUD but for other pathologies historically managed in an in-patient setting, can potentially alleviate significant pressures on stretched in-patient facilities, allowing optimal resource allocation and utilisation. Additionally, ambulatory care allows patients to maintain their daily routines, improves quality of life parameters and psychosocial well-being, and reduces the risk of ‘institutionalisation’ [30, 31]. However, patient selection is an integral and critical part of choosing patients to be managed on ambulatory pathways.

In two previous reports, surgeons and emergency physicians reported being uncomfortable using a no-antibiotic and ambulatory management strategy for AUD. Cited factors were a lack of defined hospital pathway/protocol, surgeon concerns about treatment failure, and follow-up logistics [9, 32]. 

**Fig. 2:**
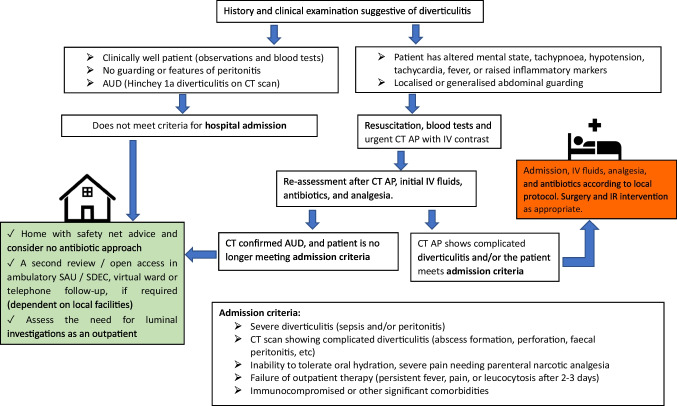
Algorithm for the management of acute uncomplicated diverticulitis (AUD) CT AP, computed tomography scan of abdomen and pelvis; IV, intravenous; SAU, surgical assessment unit; SDEC, same-day emergency care

Figure 2 shows a simple algorithm for the ambulatory management of uncomplicated diverticulitis. This pathway is currently used in one of the participating centres in the study, and was drawn from the Getting It Right First Time (GIRFT) programme. It can be adopted and tailored according to the individual hospitals’ available facilities [33].

This study provides some useful insights; however, it is essential to interpret the findings in the context of its limitations. Firstly, as a retrospective and non-randomised study, it inherently carries a risk of selection bias. The PSM analysis helps to mitigate this issue but does not completely eliminate it [34]. The other drawback of the retrospective nature of this study is the reliance on pre-existing records, which may not fully represent the entire patient population and miss relevant information. The non-standardised data collection methods used in the original records also contribute to the variability and potential errors in data collection and analysis.

Additionally, the sample size was relatively small, which limited the findings’ statistical power and may have affected the robustness of the conclusions drawn from the analysis. The effects and indications of antibiotic prescription and follow-up investigations have not been investigated thoroughly in this study due to the lack of detailed treatment protocols in most of the centres included. Lastly, the multi-centre setting of this study aids in enhancing the generalisability of the findings across different populations and settings, but it also introduces variability in the management protocols used. Different centres might have distinct treatment protocols, patient management pathways, and data recording methods, which can affect the uniformity of the data and the study’s outcomes.

## Conclusion

Our study provides evidence for the safety and effectiveness of ambulatory care in managing patients with AUD. Compared with in-patient treatment, the ambulatory pathway seems to be associated with significantly reduced hospital readmissions for AUD, with other outcomes being non-inferior. Appropriate patient selection is critical, as well as well-designed ambulatory pathways incorporating safety-netting mechanisms. This model of care has important implications for substantial cost-savings, the wider health economy, and better utilisation of limited healthcare resources.

## Supplementary Information

Below is the link to the electronic supplementary material.

ESM 1 (DOCX 32.2 KB)

ESM 2 (DOCX 164 KB)

## Data Availability

No datasets were generated or analysed during the current study.
