# Supplementary material for: Ambulatory management of acute uncomplicated diverticulitis (AmbUDiv study): a multicentre, propensity score matching study
Source: Int J Colorectal Dis. 2024 Nov 18;39(1):184. doi: 10.1007/s00384-024-04759-9 (PMC11573821; doi:10.1007/s00384-024-04759-9)
Supplement: Supplementary file 2 — (DOCX 164 KB) [file 384_2024_4759_MOESM2_ESM.docx]

1. Absolute standardised mean difference. dm, diabetes mellitus; wcc, white blood cells; crp, C-reactive protein; prevdiv, previous diverticulitis; abx, antibiotics.


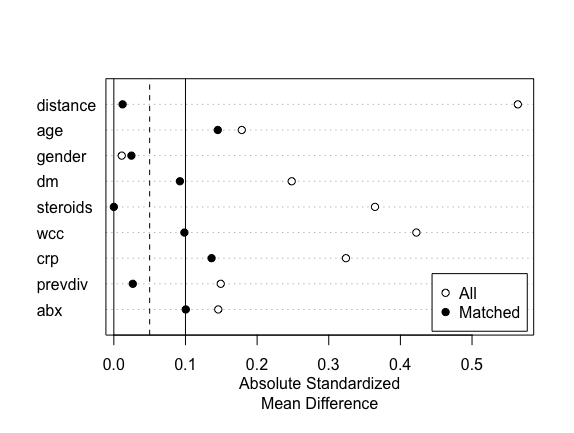


1. Covariate balance. dm, diabetes mellitus; wcc, white blood cells; crp, C-reactive protein; prevdiv, previous diverticulitis; abx, antibiotics.


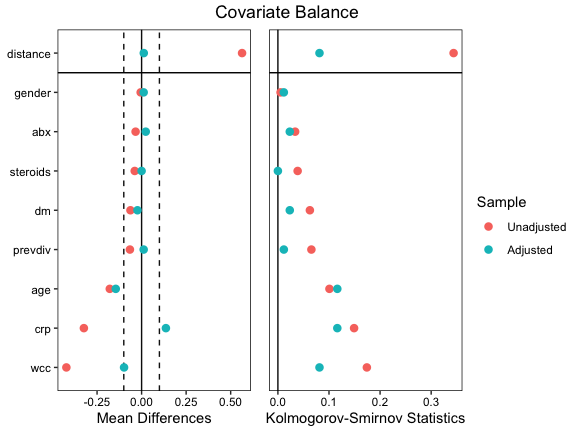


1. Distribution of propensity score.


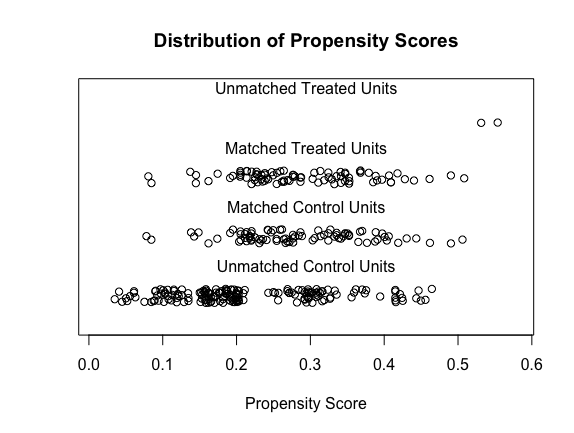


1. Histogram of unmatched and matched data


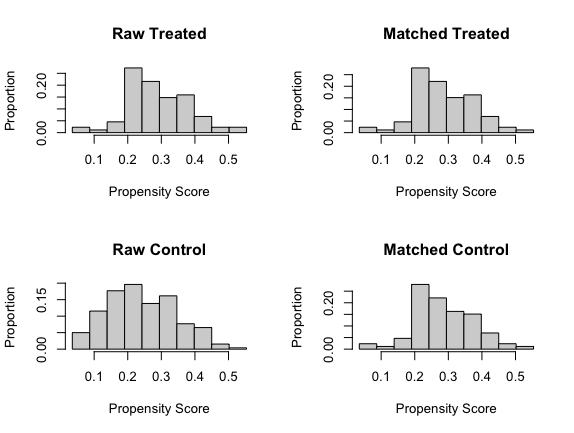


**Appendix 2:** The propensity score-matching process.
